# Implications of the South African National Drug Policy on prescribing patterns: a case study of the Limpopo province

**DOI:** 10.1186/s40545-023-00622-4

**Published:** 2023-10-02

**Authors:** Onyinye Onyeka Akunne, Vutomi Valoyi, Alexander Wehmeyer, Yasmina Johnson, Renier Coetzee

**Affiliations:** 1 School of Public Health, University of the Western Cape, Cape Town, South Africa; 2 School of Pharmacy, University of the Western Cape, Cape Town, South Africa; 3 Western Cape Department of Health, Cape Town, South Africa

**Keywords:** Medicines use, Prescribing pattern, WHO indicators, Generic prescribing, Prescribing quality

## Abstract

**Background:**

The South African National Drug Policy (SA-NDP) was introduced to promote rational medicine use (RMU). This study evaluates the quality of prescribing in the public healthcare sector in South Africa's Limpopo province following the World Health Organization's (WHO) rational prescribing standards. In addition, the prescribing practices in South Africa were compared to other African countries.

**Methods:**

A prospective cross-sectional survey of patients' prescriptions was conducted in Limpopo, South Africa, from October to December 2018. Findings were compared with the WHO reference values (WHO-RV), and the International Network of Rational Use of Drugs (IRDP) tool was used to measure the degree of rational prescribing. The optimal IRDP value was defined as 1. Study findings were compared with results from a previous study conducted in Limpopo and studies from Ethiopia and Eritrea.

**Results:**

Six hundred prescriptions were reviewed. The mean (SD) age was 43.9 ± 24.4 years (females = 56.5%). The average number of drugs prescribed (4.3, IRDP = 0.47) was higher than the WHO-RV (< 2). Generic prescribing (43%, IRDP = 0.43) and medicines prescribed from the essential medicines list (EML) (90%, IRDP = 0.90) were less than the WHO-RV (100%, respectively). Antibiotics (28%, IRDP = 1) and the number of injections prescribed (8%, IRDP = 1) were below the WHO-RV (< 30% and < 20%, respectively). The number of medicines prescribed was higher compared to previous years (4.3 vs. 3.4). Antibiotic prescribing declined (28% vs. 63.4%). Generic prescribing (43% vs. 41.7%) and medicines prescribed from the EML (90% vs. 93.1%) did not improve. A higher number of medicines were prescribed in this study compared to Ethiopia (1.7) and Eritrea (1.8), and a lower number of antibiotics were prescribed compared to Ethiopia (58.2%) and Eritrea (54.5%). Generic prescribing was low compared to Ethiopia (95.6%) and Eritrea (94.9%). All studies showed reduced injection prescribing (6.6–15.9%) and similar prescribing from the EML (90–95%).

**Conclusions:**

There is an increased potential for drug-drug and adverse reactions with the increased number of prescribed medicines; however, the patient's clinical needs may warrant using multiple medicines. There is a need for generic prescribing to reduce medicine expenditure.

## Introduction

Rational medicine use requires that patients receive medications appropriate to their clinical needs, in doses that meet their own individual requirements, for an adequate period of time, and at the lowest cost to them and their community [1]. In line with the World Health Organization (WHO) initiative for rational medicine use, the National Drug Policy (NDP) of South Africa published in 1996 aims to promote the rational use of medications, ensure quality dispensing and prescribing practices, lower medicine costs as well as promote cost-effective prescribing [2]. Evaluating change in prescribing practices in South Africa since implementing the NDP is essential to ensure prescribers follow the best practices.

The World Health Organization (WHO), in collaboration with the International Network of Rational Use of Drugs (INRUD), established a standardized tool to measure rational medicine use. This tool assesses the number of medicines prescribed to patients, percentages of generic, antibiotic, injectable medicines, and medicines prescribed from the essential medicines list (EML) [3]. Irrational prescribing has been demonstrated to increase medicine expenditure and negatively impact the quality of patients' care. Irrational prescribing includes overprescribing medicines, prescribing antibiotics without sufficient evidence of an infection, inappropriate use of injections, prescribing medicines by their brand names, and not adhering to established EMLs [4]. Rational prescribing is particularly important to patients with chronic illnesses as their continued adherence to their regimen is critical to managing their disease condition.

A South African-based study conducted at private medical practices and public hospitals in Limpopo and Western Cape provinces aimed to examine medicine prescribing quality using the WHO prescribing indicators. The study showed an elevated number of antibiotic prescriptions (63.4% and 72.8%, respectively) and fewer medicines prescribed by their generic names (41.7% and 48.6%, respectively) in public healthcare facilities [5].

Further, the study demonstrated that not all medications prescribed during the study period were according to the EML [5]. Prescribing an antibiotic when it is not warranted may lead to the emergence of antibiotic resistance, which could have life-threatening effects on the population. It has been well-established that patient adherence is diminished when numerous medicines are prescribed and when generic prescribing is low [6].

This study aims to evaluate the quality of prescriptions in the Limpopo province of South Africa using the WHO-INRUD indicators, and consider the prescribing changes over time. In addition, study findings were compared with findings from other African countries.

## Methods

### Study design

This study was a retrospective cross-sectional survey of patients' prescriptions from October to December 2018. The WHO/INRUD guideline and standardized data collection tool were used for data collection. The guideline recommends the selection of 30 patients' prescriptions from 20 facilities to evaluate rational medicine use [3].

### Study location

The study was carried out at district and regional hospitals in the Limpopo province, one of the nine provinces in South Africa. The Limpopo province comprises five districts with 39 district hospitals and six regional hospitals. The District hospitals typically have 50 to 600 beds, whilst regional hospitals have 200 to 800 beds, depending on the size of the hospital. Four hospitals per district were conveniently selected to ensure a representative sample.

### Inclusion criteria

The inclusion criteria for patients were (i) patients of all ages, (ii) patients with a prescription available (iii). Patients who are willing to participate in the study.

### Exclusion criteria

The exclusion criteria for patients included (i) patients with human immunodeficiency virus (HIV) or tuberculosis (TB) due to the associated high pill burden in these populations, which could confound the results, (ii) Patients not willing to participate in the study.

### Data collection

Patients' prescriptions were collected from the pharmacy after patients' visits on specified data collection days. The patient prescriptions were randomly selected.

Patient age, gender, and the number of medicines prescribed, including injections and antibiotics, were collected from the prescriptions and captured into pre-developed collection tools, which included the WHO prescriber indicators tools. The indicators evaluated included the average number of medicines per encounter, the percentage of generic medicines prescribed, the percentage of antibiotics prescribed, the percentages of injections prescribed and the percentage of medicines prescribed according to the relevant EML.

### Data analysis

SPSS software version 25.0 was used to analyze the data. The quality of medicine prescribing was measured using the WHO/INRUD optimal prescribed levels indices [7]. The degree of rational prescribing was measured using the Index of Rational Drug Prescribing (IRDP), validated and utilized by Dong et al. [8]. This index was also employed in the study conducted by Atif et al. [9]. The optimal IRDP value for each WHO/INRUD prescribing indicator was set at 1, with values exceeding 1 considered as 1. Values found to lie closer to or at one were considered to have had greater rational prescribing than indicators closer to or at 0. The IRDP for non-polypharmacy (reduced average number of medicines per prescription), rational antibiotics used and safe injection use was calculated as follows:

$$\frac{optimal \,(reference) \,value}{observed \,value}$$

The IRDP for generic prescribing and prescribing from the EDL was calculated as follows:

$$\frac{observed\, value}{optimal\, (reference) \,value}$$

This study compared prescription indicator values to a study that investigated prescribing quality in both the Limpopo and Western Cape provinces of South Africa in 2005 [5]. This comparison was utilized to evaluate for possible changes in medicine prescribing over time. Studies in Africa conducted during the same period as this study (2018) were identified [10, 11], and their results were also compared to those of this study.

## Results

Six hundred patient prescriptions from 20 hospitals in the five districts were reviewed. The mean (SD) age of patients was 43.9 ± 24.4 years. The ratio of males (N = 261, 43.5%) to females (N = 339, 56.5%) was 0.7:1. Table 1 shows the age and gender distribution of patients across the 20 facilities.

**Table 1:** Age and gender distribution of patients attending the twenty facilities included in the study

Facility number	Average age (years)	Minimum age (years)	Maximum age (years)	Gender	
				Male (%)	Female (%)
1	48.73	3	95	9 (30.00)	21 (70.00)
2	42.07	3	89	14 (46.67)	16 (53.33)
3	45.40	5	88	13 (43.33)	17 (56.67)
4	49.20	1	86	13 (43.33)	17 (56.67)
5	42.97	1	88	15 (50.00)	15 (50.00)
6	35.37	1	79	14 (46.67)	1 (53.33)
7	47.40	4	96	9 (30.00)	21 (70.00)
8	52.97	1	98	12 (40.00)	18 (60.00)
9	49.10	21	79	13 (43.33)	17 (56.67)
10	50.23	3	86	15 (50.00)	15 (50.00)
11	43.97	1	103	16 (53.33)	14 (46.67)
12	48.93	9	79	19 (63.33)	11 (36.67)
13	37.13	1	99	18 (60.00)	12 (40.00)
14	36.60	4	85	6 (20.00)	24 (80.00)
15	49.13	2	99	14 (46.67)	16 (53.33)
16	38.00	1	84	9 (30.00)	21 (70.00)
17	37.07	3	51	14 (46.67)	16 (53.33)
18	48.47	1	86	14 (46.67)	16 (53.33)
19	42.3.0	1	90	9 (30.00)	21 (70.00)
20	32.00	1	69	15 (50.00)	15 (50.00)
Total age (SD)	43.85 (24.42)	1	103	261 (43.5%)	339 (56.5%)

The average number of medicines per encounter was 4.3, more than twice that of the reference value (Table 2). Prescribers used generic names for less than 50% of the medications prescribed. Ninety percent of medicines prescribed were prescribed according to the EML. Antibiotics and injections were found to be prescribed within the limits of the designated reference values (< 30% and < 20%, respectively). The IRDP was optimal (IRDP = 1) for both the number of antibiotics and injections prescribed and was elevated for the prescription of medicines according to the EML (0.90). Low IRDP values were observed for non-polypharmacy (0.47) and generic prescribing (0.43).

**Table 2:** Quality of medicines prescription in the Limpopo healthcare facilities

WHO prescribing indicator	Reference value [8]	Study value	IRDP
The average number of medicines per encounter	< 2	4.3	0.47
% medicines prescribed by generic name	100%	43%	0.43
% encounters with an antibiotic prescribed	< 30%	28%	1
% encounters with an injection prescribed	< 20%	8%	1
% medicines prescribed from an essential medicines list or formulary	100%	90%	0.90

A comparison of these results with that of the study examining prescribing patterns in South Africa in 2005 revealed an upward trend in the total number of medicines prescribed. However, the number of antibiotics prescribed was reduced by more than 50%. Generic prescribing was similar, with less than half of medications prescribed by their generic names. The prescribing of injections remained low at less than 10%. Approximately 10% of the medicines prescribed were not on the EML (Table 3).

**Table 3:** Medicines prescription patterns in selected South African provinces over time

WHO prescribing indicator	Limpopo Oct–Dec 2018*	Limpopo Aug–Dec 2005**	Western Cape Aug–Dec 2005**
The average number of medicines per encounter	4.3	3.4	3.0
% Medicines prescribed by generic name	43%	41.7%	48.6%
% Encounters with an antibiotic prescribed	28%	63.4%	72.8%
% Encounters with an injection prescribed	8%	9.8%	6.7%
% Medicines prescribed from an essential medicines list or formulary	90%	93.1%	92.0%

*Study value; **Data from public healthcare clinics[5]

The average number of medicines per prescription in South Africa was more than 100% of the average number of medicines per prescription in Ethiopia and Eritrea during the same study period. Generic prescribing was markedly less in South Africa (43%) compared to both Ethiopia (95.6%) and Eritrea (94.9%), respectively. Antibiotic prescribing was less in South Africa (< 30%) compared to Ethiopia (58.2%) and Eritrea (54.5%). The percentage of prescriptions with an injection prescribed was low (< 20% advocated) in all countries. Prescribing medicines from the EML was between 90 and 95% in all three countries (Table 4).

**Table 4:** Comparison of medicines prescription patterns across three African Countries

WHO prescribing indicator	South Africa Oct–Dec 2018*	Ethiopia Jan 2017–June 2019**	Eritrea Sept 2017–Jan 2018***
The average number of medicines per encounter	4.3	1.7	1.8
% Medicines prescribed by generic name	43%	95.6%	94.9%
% Encounters with an antibiotic prescribed	28%	58.2%	54.5%
% Encounters with an injection prescribed	8%	15.9%	6.6%
% Medicines prescribed from an essential medicines list or formulary	90%	93.9%	94.8%

*Study values, **Tassew et al. 2021[11], ***Siele et al. 2022[10]

## Discussion

The study findings indicate that the number of medicines per prescription has increased in South Africa. Furthermore, it was identified that this value was more than double that reported by other African countries [10, 11]. There is a more significant potential for drug-drug, drug-herb and drug-food interactions and an increased risk of adverse effects with more prescribed medications. Adverse effects of medicines can affect patients' adherence to their treatment, which is particularly significant in chronic illness as optimal disease management is mainly dependent on adherence [12].

Irrational prescribing includes prescribing medicines that are not required by patients either because there is no evidence-based indication, the intended therapeutic benefit is not derived from the treatment, the medicine places the patient at an elevated risk of adverse effects, or the patient is not willing or able to administer the medicine as prescribed [12]. Although the number of medicines prescribed in this study was more significant than that recommended by the WHO [8], the appropriateness of medications according to indication and the outcome of therapy, including the resolution or management of diseases, was not evaluated. Therefore, these findings could not evaluate the appropriateness of the medicines prescribed.

Generic prescribing was low, with less than 50% of the medicines evaluated being prescribed by their generic name. Generic medicines boast a greater level of cost-effectiveness compared to innovator brands, and their prescription is advocated to reduce medicines expenditure, especially when patients purchase their medication with out-of-pocket payments. Yang et al. (2020) conducted a study to examine how payment and prescription-related factors affected antihypertensive adherence in the United States of America (US). The study revealed that prescribing generic medicines improved patients' adherence to their regimens [6]. The key economic objectives of the South African NDP include improving rational medicine use, decreasing medicine costs, and promoting cost-effective medicine prescribing [13]. Therefore, it should be recognized that prescribing generic medications can reduce the cost of medications. Despite the NDP being promulgated approximately 30 years ago, an optimal level of generic prescribing has not been achieved.

Comparing the % of generic prescribing in this study with baseline values (36%) from a study conducted between 1996 and 1998, just after the NDP was published, reveals that the policy has not successfully enhanced generic prescribing [14]. These findings reveal gaps in the implementation of the NDP in South Africa and accentuate the need for further studies investigating the impact of these gaps on health outcomes.

The effective antibiotic prescription observed in this study could result from greater acceptance and incorporation of antimicrobial stewardship programmes in the country's healthcare facilities over the last two decades [15, 16]. These programmes must be assessed and updated regularly to maximize and maintain their performance.

Injection prescriptions in this study were low, and this finding is reflected in previous findings in South Africa and other parts of Africa [5, 10, 11]. Over the years, increased awareness of the likelihood of disease transmission with injection use may have played a crucial role in the decline observed and increased substitution of IV with oral medicines.

Prescribing from the EML was high in this study but not 100%. A recent study in South Africa showed lower prescribing from the EDL compared to this study [17]. Prescribing from the EML improves rational prescribing as the medicines list was compiled according to the best evidence and considers the cost-effectiveness of the treatment.

## Conclusion

The quality of prescribing was optimal for two indicators—the percentage of antibiotics and injections prescribed. Constant monitoring and improvement of the systems enhancing this high quality should be established to maintain rational antibiotic and injection prescribing. Prescription of medicines from the EML was high but not at the optimal 100% level. Interventions to improve prescribing from the EML, including frequent updates of the EML to cater to the prescribers' concerns, will improve rational medicine use. Generic prescribing was consistently low, and measures to improve the prescription of medicines by their generic name should be implemented. The number of medicines per prescription was high, but the effect on clinical outcomes needs to be evaluated. There is a need for stakeholders to work together to improve rational prescribing collectively. Prescribers should try as much as possible to minimize the number of medications prescribed to patients. Prescribing fixed-dose combinations may improve patients' adherence to treatment as the number of medicines is reduced. Pharmacists have a role in identifying irrational prescriptions and collaborating with prescribers to make corrections.

## Data Availability

The data and materials used are available upon reasonable request.

## Abbreviations

ADR: Adverse drug reaction
EML: Essential medicines list
HIV: Human immunodeficiency virus
INRUD: International Network of Rational Use of Drugs
IRDP: Index of Rational Drug Prescribing
NDP: National Drug Policy
SA-NDP: South African National Drug Policy
TB: Tuberculosis
WHO: World Health Organization
WHO-RV: World Health Organization reference values
